# Beyond yields: a systems approach is essential for reconciling agriculture and biodiversity

**DOI:** 10.1098/rstb.2025.0257

**Published:** 2025-08-14

**Authors:** Damien Beillouin, Sarah K. Jones, Bruno Rapidel, Natalia Estrada-Carmona

**Affiliations:** ^1^ UPR HortSys, CIRAD, 34398 Montpellier, France; ^2^ CIRAD, University of Montpellier, 34398 Montpellier, France; ^3^ Bioversity International, 34397 Montpellier, France; ^4^ ABSys, University of Montpellier, 34090 Montpellier, France

**Keywords:** biodiversity, land use, farming, yield, food production

*Phil. Trans. R. Soc. B* **380**: 20250258 (Published online 07 August 2025). (https://doi.org/10.1098/rstb.2025.0258)

## 1. Introduction

Balmford *et al*. [1] advocate land sparing and high yields as the primary strategy to address biodiversity loss and ensure food security. We challenge the notion that managing agricultural land solely to optimize yields can halt or reverse the decline of biodiversity [2]. Decades of yield-centric agriculture, divorced from ecological and social concerns, have proven inadequate.

## 2. Rethinking agricultural productivity beyond yields

Despite acknowledging agriculture’s profound negative impacts on biodiversity and ecosystem function, the authors advocate ‘sustainable high-yield agriculture’ primarily through ‘*sustained access to fertilizer, improved varieties* [*including genetically modified*]*, markets and sound agronomic advice*’. These practices are similar to those promoted under the Green Revolution’s tech-driven strategies [3, p. 8], raising concerns about how their sustainable model truly differs from the current high-input agricultural intensification paradigm. This approach risks perpetuating biodiversity decline [4], agriculture’s contribution to a quarter of global greenhouse gas emissions [5], inadequate remuneration of farmers [6], and malnutrition affecting at least 735 million people [7]. Agrochemical dispersal degrades ecosystem functions at regional and global scales [8]. Intensive agriculture perturbs hydrological cycles, exacerbating water scarcity and driving planetary boundary transgressions related to freshwater and land-system change [9]. Moreover, atmospheric nitrogen deposition, a result of fertilizer use, alters distant ecosystems, impacting global biodiversity and biogeochemical flows [10]. A singular focus on maximizing yields, ignoring environmental and social externalities—inadequately addressed in their text—risks exacerbating the hidden costs of food systems, estimated at $10 trillion in purchasing power parity in 2020 [11]. Notably, 70% of those costs concern human health, followed by environmental degradation. From a policy perspective, high-yield farming is economically questionable, since many associated costs are partially covered through highly distortive, environmentally and socially detrimental public agriculture support mechanisms [12]. These direct subsidies, input-based subsidies and export subsidies deplete public funding that could support education, healthcare and biodiversity conservation. By ignoring high-yield industrial systems’ structural limitations, technocratic approaches could limit solutions to partial fixes and overlook alternatives rooted in sustainability, equity and resilience.

We argue sustainable agriculture should be defined by its proven ability to deliver and ensure access for everyone to healthy foods, while maintaining/improving natural resources and ensuring the wellbeing of all farmers. Context-specific adaptation and continuous scientific evaluation beyond high yield or organic practices are essential, as advocated in the agroecology literature [13]. For example, agroecology prioritizes optimizing whole-farm and landscape socio-ecological principles contributing to soil health, biodiversity, resource circularity, justice, equity and access to affordable, locally sourced and nutritious food [14]. Balmford *et al.* exhibit a paradoxical stance, acknowledging briefly the potential of ‘*less capital-intensive approaches*—*including integrated pest management, push–pull methods of controlling pests, co-culture techniques, silvopasture and drip irrigation—to achieve marked increases in yields, often with lower inputs of water or potentially harmful chemicals*’ [1, p. 8], yet later implying an inherent trade-off between yield and environmental integrity within agroecological systems (see ‘*The chief challenge to land sharing is that most actions ... typically tend to reduce farm yields*’ [1, p. 5]). This assumption is challenged by empirical evidence suggesting that such trade-offs are not inevitable [15], and that agroecological practices, including agroforestry and cover cropping, can simultaneously enhance agricultural productivity and profitability, improve ecosystem services and contribute to food security [16–22] in a changing climate [23].

## 3. The need for on-farm biodiversity conservation to achieve the world’s biodiversity goals

Rather than improving the state of the world’s biodiversity, we argue that relying solely on high-yield intensive agriculture for food production will compromise achieving Global Biodiversity Framework (GBF) targets 1, 4 and 10 [24] and multiple Sustainable Development Goals [25]. Balmford *et al*.’s advocacy for land sparing is undermined by three critical oversights: a flawed assumption of effective ‘spared’ land protection, an underestimation of leakage effects, and a neglect of agrobiodiversity’s crucial role.

First, Balmford *et al*.’s land-sparing paradigm, reliant on assumed effective protection, is frequently contradicted by empirical evidence. Effective ‘spared’ land protection necessitates more than spatial designation; it requires legally binding protection, local community engagement and ensuring that ‘spared’ areas form part of a functional ecological network. Without these, spared lands are susceptible to fragmentation [26], disturbance and conversion, including from distant but powerful drivers of land use change such as illegal logging, urbanization, and infrastructure development [27]. While protected areas have successfully contributed to reducing deforestation and improving livelihoods, their effectiveness in achieving broader conservation goals is contested in certain contexts [28,29].

Second, Balmford *et al*.’s analysis of the Jevons paradox potentially underestimates the substantial impacts involved (cf. ‘Jevons effects are rare’ [1, p. 9]) [30,31], while the discussion on leakage is incomplete. The authors acknowledge spatial displacement of agricultural production, but neglect the equally critical phenomenon of consumption leakage, i.e. efficiency gains leading to increased consumption. Increased agricultural productivity, even when spatially confined, can precipitate lower food prices, thereby stimulating heightened demand and consumption, ultimately driving further land conversion beyond initially spared areas [32]. Balmford *et al.* link agriculture’s ecological footprint to consumption, trade, and supply chains [33]. They suggest that the European Union (EU)’s Biodiversity and Forest Strategies for 2030 requirement to spare old-growth forests and reduce yields in other forests is undesirable, on the basis that sparing tropical forests is more important for biodiversity. Yet, biodiversity needs conserving in every biome [34]. They also overlook the EU Regulation on Deforestation-free Products which is designed to mitigate leakage effects by requiring uniform forest protection rules.

Finally, the authors overlook the risks posed by genetic erosion, an inherent consequence of intensive high-yield farming systems [24,35]. This erosion represents a systemic loss of adaptive capacity, reducing the potential for future agricultural innovation and therefore threatening long-term food security. GBF targets 4 and 10 highlight the need to include agrobiodiversity—such as underutilized varieties and breeds and species like pollinators and soil organisms—in conservation to protect them from extinction while supporting agriculture [24]. Safeguarding this biodiversity requires consuming neglected foods and restoring ecosystem functioning through integrated spatial planning (GBF target 1) [36], efforts at risk from a narrow focus on maximizing yields.

## 4. Ensuring a socially just approach

The authors’ advocacy for land sparing overlooks critical socio-ecological complexities of non-integrated land-use planning, farmer decision-making and agricultural transitions.

First, promoting large-scale intensification of agricultural land could reinforce agribusiness dominance of land and food systems, while marginalizing smallholder farmers and Indigenous peoples [30], and increasing local inequalities [37]. Technology-based intensification is capital-intensive, which smallholders struggle to compete with, leading to displacement or economic dependence on large agribusinesses [38]. A key example of this dynamic is the promotion of genetically modified organisms. Despite promises of higher yields, they have failed to address food security in regions with significant yield gaps, particularly in Africa [39]. The ban of Bt cotton in Burkina Faso due to performance issues [40], mixed outcomes in India [41], and loss of farmer seed sovereignty [42] highlights the limits of over-reliance on this corporate-driven approach [43]. As acknowledged in the agroecology literature [44] and beyond [45], achieving ‘sustainable’ agriculture requires that agricultural landscapes are recognized and managed as the multifunctional systems that they are [32].

Second, the authors over-simplify decision-making processes, ignoring evidence of the multi-criteria determinants of adoption and maintenance of ecologically friendly practices by farmers [46]. These studies show cultural values, knowledge access, social networks, land security and awareness of long-term ecological benefits shape farmers' decisions. Likewise, the authors condemn input reduction efforts by relying on a poorly planned transition example (the Sri Lanka case) while ignoring successful efforts aiming to reduce chemical use and dependency, such as the regulated markets for diversified family farming in Brazil [47], voluntary participation schemes for organic farming in Cuba [48] and government-supported natural farming in India [49] .

In conclusion, a yield-centric approach to agriculture risks reinforcing the prioritization of ‘short-term, individual, and material gains’ [4, p. 12] that drive biodiversity loss, climate change and pollution. Addressing climate change, habitat loss and pollution requires transformative change beyond incremental land-use efficiency gains [4]. For this, we call on science to empirically validate which farming practices, agrifood system structures and transition pathways are capable of providing the world with a socially just, biodiverse, climate-, water- and food-secure future.

## Data Availability

This article has no additional data.
